# Age-Related Modulation of the Effects of Obesity on Gene Expression Profiles of Mouse Bone Marrow and Epididymal Adipocytes

**DOI:** 10.1371/journal.pone.0072367

**Published:** 2013-08-14

**Authors:** Li-Fen Liu, Wen-Jun Shen, Masami Ueno, Shailja Patel, Salman Azhar, Fredric B. Kraemer

**Affiliations:** 1 Division of Endocrinology, Stanford University, Stanford, California, United States of America; 2 Veterans Affairs Palo Alto Health Care System, Palo Alto, California, United States of America; Wayne State University, United States of America

## Abstract

This study aimed to characterize and compare the effects of obesity on gene expression profiles in two distinct adipose depots, epididymal and bone marrow, at two different ages in mice. Alterations in gene expression were analyzed in adipocytes isolated from diet-induced obese (DIO) C57BL/6J male mice at 6 and 14 months of age and from leptin deficient mice (*ob/ob*) at 6 months of age using microarrays. DIO affected gene expression in both depots at 6 and 14 months, but more genes were altered in epididymal than bone marrow adipocytes at each age and younger mice displayed more changes than older animals. In epididymal adipocytes a total of 2789 (9.6%) genes were differentially expressed at 6-months with DIO, whereas 952 (3.3%) were affected at 14-months. In bone marrow adipocytes, 347 (1.2%) genes were differentially expressed at 6-months with DIO, whereas only 189 (0.66%) were changed at 14-months. 133 genes were altered by DIO in both fat depots at 6-months, and 37 genes at 14-months. Only four genes were altered in both depots at both ages with DIO. Bone marrow adipocytes are less responsive to DIO than epididymal adipocytes and the response of both depots to DIO declines with age. This loss of responsiveness with age is likely due to age-associated changes in expression of genes related to adipogenesis, inflammation and mitochondrial function that are similar to and obscure the changes commonly associated with DIO. Patterns of gene expression were generally similar in epididymal adipocytes from *ob/ob* and DIO mice; however, several genes were differentially expressed in bone marrow adipocytes from *ob/ob* and DIO mice, perhaps reflecting the importance of leptin signaling for bone metabolism. In conclusion, obesity affects age-associated alterations in gene expression in both epididymal and bone marrow adipocytes regardless of diet or genetic background.

## Introduction

Obesity increases the risk and prognosis of many diseases, including type 2 diabetes and cardiovascular disease [1]. The type and distribution of fat contribute to increased risk for insulin resistance. Intra-abdominal fat, referred to as “visceral” fat, accounts for a relatively small proportion (15%) of total body fat mass; yet, it appears to correlate more highly with insulin resistance than does subcutaneous fat mass [2,3].

Several studies have reported the gene expression differences between adipose tissue obtained from different depots in rodents and humans [4,5]. The capacity of preadipocytes obtained from visceral depots to undergo adipogenesis is less than that taken from subcutaneous adipose tissue in obese subjects [6,7]. A reduced adipogenic/lipogenic expression is correlated with hepatic steatosis and insulin resistance in obesity [8]. It has been reported that there are differences in the responses of adipose depots to overfeeding in humans, suggesting that inherent differences in preadipocytes might contribute to the different responses of fat depots to overfeeding and obesity [9].

Microarray analysis of epididymal adipose tissues taken from lean, obese and obese-diabetic mice demonstrated differential adipocyte-specific gene expression [10]. While a number of studies have examined potential metabolic differences between adipose depots, i.e., visceral versus subcutaneous, few studies have examined bone marrow adipose cells and how they respond to obesity. Moreover, no studies have examined the impact of age on the response of adipose cells to obesity. We have reported that bone marrow adipocytes represent a unique adipose depot with low expression of adipose-specific genes and high expression of genes associated with adipose differentiation, as well as with inflammation, compared with epididymal adipose cells [11]. A similar gene expression profile was also reported in bone marrow progenitor derived adipocytes in mice [9,12]. Interestingly, the impact of age on gene expression of bone marrow and epididymal adipose cells differs. For example, genes associated with adipose differentiation are increased with age in bone marrow adipose cells, but decrease with age in epididymal adipose cells [11]. Bone marrow adipose cells are metabolically active, but, while their functional significance is not fully established [13], there is a close link between bone remodeling and metabolic homeostasis through a novel endocrine loop consisting of leptin, osteocalcin and insulin [14,15]. Thus, understanding the differences between bone marrow and epididymal adipocytes in response to obesity could provide important information towards understanding the role of bone marrow adipocytes.

The aim of present study was to characterize and compare the effects of diet-induced obesity (DIO) on gene expression profiles in two distinct adipose depots, epididymal and bone marrow adipocytes, at two different ages, as well as compare these depots in *ob/ob* mice, a genetic model of obesity due to leptin deficiency. Microarray analysis was conducted to examine the effect of obesity on gene expression of epididymal and bone marrow adipocytes within the same animal and to characterize the differences in gene expression between DIO mice and *ob/ob* mice. Our results demonstrate that bone marrow adipocytes, although responsive, are less responsive to a high fat diet than epididymal adipocytes and the response of both adipocyte depots to a high fat diet declines with age.

## Methods and Procedures

### Ethics Statement

All procedures involving animals were in accordance with institutional and national guidelines and approved by the Institutional Animal Care and Use Committee of the VA Palo Alto Health Care System.

### Experimental animals and diets

Male C57BL/6J mice aged 3-months and 11-months and leptin deficient (*ob/ob*, 6-months) male mice were purchased from Jackson Laboratory (Bar Harbor, ME, USA). After 1 week of acclimation, lean mice were fed either with a standard chow (10% of total calories from fat) or a high-fat diet (HFD, 60% of total calories from fat, D12492 Research Diets, Inc, NJ, USA) for 12 wks prior to sacrifice at 6 and 14 months. Detailed diet composition can be found at the manufacturer’s website researchdiets.com. All mice were housed in temperature-controlled conditions on a 12-h light, 12-h dark cycle.

### Histology

Distal femurs isolated from 6-month-old and 14-month-old mice were decalcified in 4% EDTA and paraffin embedded following the manufacturer’s standard procedures (Histion, Everett, WA, USA). Bones were sectioned in the sagittal plane to obtain cross sections of the distal femur and stained with hematoxylin and eosin (H&E). Fields were taken from distal femur sections of 6-month-old and 14-month-old mice and calculated using ImagePro software.

### Isolation of primary adipocytes

Bone marrow adipocytes and epididymal adipocytes (n=6-10 animals per group) were isolated from mice. Briefly, both femurs and tibias were collected after the mice were sacrificed. Bones were cleaned and rinsed with 75% ethanol and DEPC water to eliminate surrounding fat and muscle cells. Fresh bone marrows from femurs and tibias were flushed out with PBS containing 1% fatty acid-free BSA and 1% RNAase and DNase-free water using a 25-gauge needle. Red blood cells were lysed using red cell lysis buffer. After centrifugation at 3000 RPM for 5 min, floating adipocytes were separated from bone marrow stromal cells and then were washed with PBS buffer three times. Epididymal adipocytes were isolated as described previously [16]. Briefly, epididymal white adipose tissue (WAT) was removed from mice and minced with scissors into 2 ml Kreb-Ringer HEPES buffer supplemented with 3% BSA. Tissues were digested with collagenase type I (1mg/ml) for 40 min at 37^°^ C in a 150 rpm shaker and adipocytes then isolated by flotation.

### Determination of insulin and adipokine concentrations by Luminex ELISA

Serum obtained from mice was analyzed for adipokines and bone panel measurements using a multiplex mouse adipokines assay (Mouse Adipocyte Panel, Millipore, Bedford, MA, USA), and detected by Luminex xMAP (Luminex 200, Millipore, Bedford, MA, USA). Insulin and adipokines including adiponectin, leptin, resistin, and bone markers osteocalcin, RANKL and osteoprotegerin were measured in 6-month, 14-month-old lean, HFD, and *ob/ob* mice in the fed state.

### Microarray analysis

#### RNA isolation, purification and array procedures

Total RNA was extracted using Trizol (Life Technologies, Grand Island, NY, USA) and chloroform followed by purification on an RNeasy MiniElute column (QIAGEN, Valencia, CA, USA). Three pooled RNA preparations were generated from 6–10 animals due to the low yield of bone marrow adipocytes during isolation. RNA quality was verified using an Agilent Bioanalyzer (Agilent technologies, Palo Alto, CA, USA). Total RNA was biotin-labeled and hybridized to the GeneChip Mouse Gene 1.0 ST Array platform (Affymetrix, Santa Clara, CA, USA) with three RNA preparations per age group. The Protein and Nucleic Acid Microarray Facility at Stanford University carried out processing of DNA arrays according to standard protocols from the Affymetrix GeneChip® Whole Transcript Sense Target Labeling Assay. This assay is designed to generate amplified and biotinylated sense-strand DNA targets from the entire expressed genome without bias. This assay and associated reagents have been optimized specifically for use with the GeneChip® ST Arrays where “ST” stands for “Sense Target” and the probes on the arrays have been selected to be distributed throughout the entire length of each transcript. The microarray data files have been submitted to the Gene Expression Omnibus (GEO); the accession number is GSE27017.

#### Statistical analysis

The raw data from microarrays were analyzed using Partek^®^ Genome Suite software, version 6.3 Copyright © 2008 (Partek Inc., St. Louis, MO, USA). Briefly, Affymetrix. CEL files were processed to generate gcRMA (robust multi-array average) values. This step was followed by quantile normalization and log_2_ transformation to represent gene expression levels. Samples were grouped into cell types (bone marrow adipocytes vs. epididymal adipocytes), diet (standard chow vs. HFD) and age (6-month (6M), 14-month (14M)). Three-way ANOVA was performed including, diet, age and cell type interaction to generate the lists of differentially expressed genes comparing bone marrow adipocytes with epididymal adipocytes in response to HFD and age. There were three gene chips for each group. A total of 30 individual arrays contributed to the analyses including DIO C57BL/6J male mice and leptin-deficient *ob/ob* mice. For the comparison between bone marrow and epididymal adipocytes, probe sets with a fold-change 2.0 and adjusted *p*-value <0.05 were considered differentially expressed between two cell types in *ob/ob* mice. For the comparison between standard chow and HFD at each age group, the analyses were set with a fold change 2.0 and adjusted *p*-value <0.05. The Benjamini-Hochberg false discovery rate (FDR) method was used for false positives. A corrected p-value cutoff of 0.05 was used to select the regulated genes with the lowest FDR. Partek^®^ Genome Suite was used as the first step for quality control (QC) of the data on all the samples with two methods, Pearson correlation and Principal Component Analysis (PCA). PCA was performed as a global view of sample clustering, which is related to the total variance in gene expression for all genes. Normalized expression values for all genes were analyzed.

Statistical analysis of metabolic parameters was performed using Graphpad Prism 4.0. Diet-dependent changes were statistically analyzed by two-way ANOVA (repeated measures for within subject samples, Tukeys test for post-hoc analysis).

#### Pathway Analysis

For each comparison a list of differentially expressed genes was generated. The gene lists, along with associated expression or fold-change values, were further analyzed using Ingenuity Pathway Analysis (Ingenuity system, Inc, Redwood City, CA, USA) to identify differentially expressed pathways that are affected by HFD in both bone marrow and epididymal adipocytes at each age. The list of significantly regulated genes selected by the microarray analysis described above was loaded in IPA with the following criteria: reference set: Mouse 1.0 ST Gene assay; direct and indirect relationships included filtered by species (mouse) and by tissue (adipose). Then IPA computed the data to generate significant networks of genes that are associated with particular biological functions, diseases and signaling pathways.

## Results

### Metabolic characteristics

**Figure 1:**
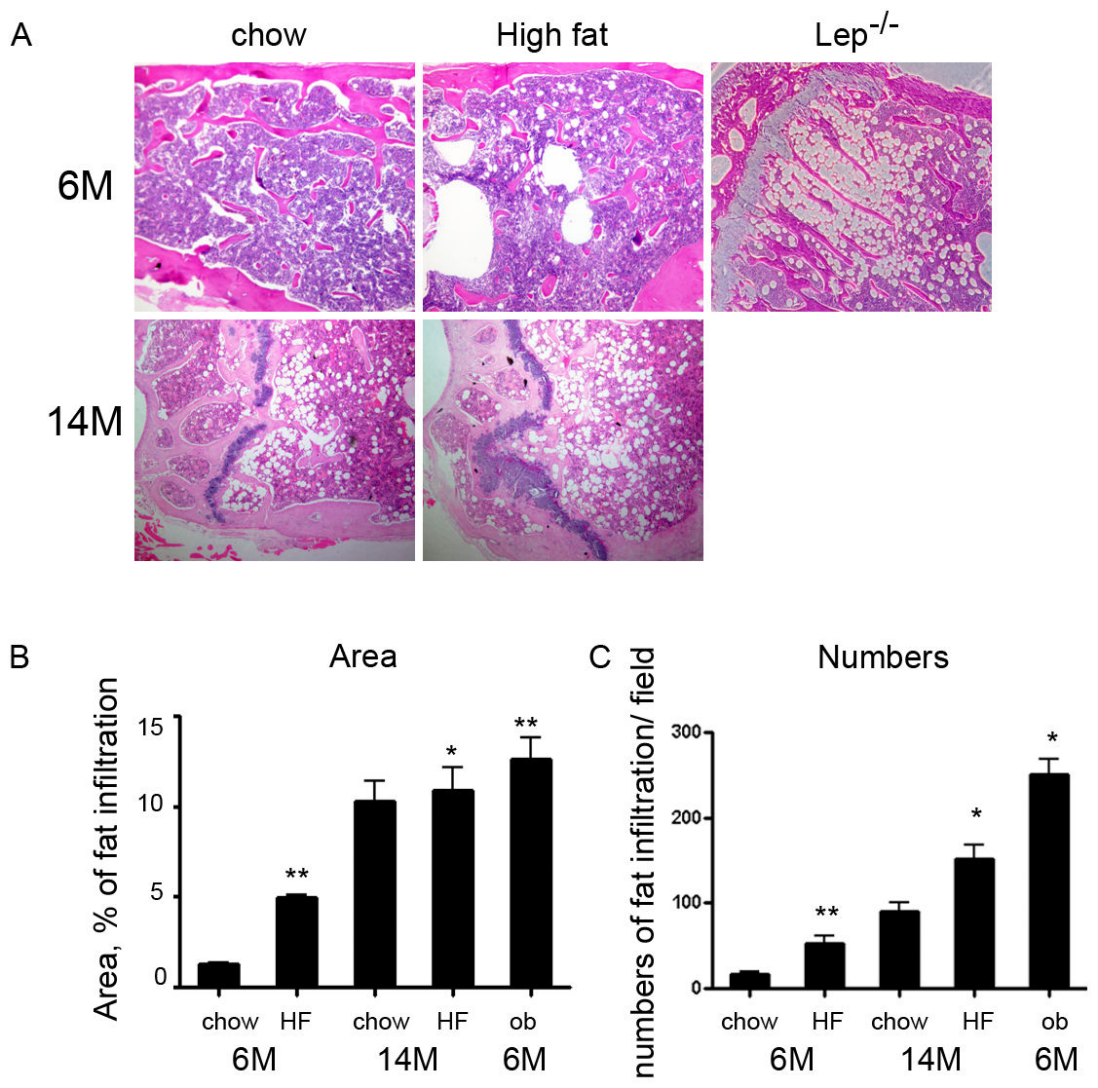
Quantitative analysis of bone marrow adipocytes. (a) Histology of the distal femurs of mice stained with hematoxylin and eosin. The top panels are from 6-month-old C57BL/6J male mice fed standard chow or high fat diet, and from 6-month-old *ob/ob* mice. The bottom panels are sections of distal femurs stained with hematoxylin and eosin from 14-month-old mice fed either standard chow or high fat diet. (b) Area of bone marrow fat infiltration as a % of total area. (c) Numbers of bone marrow fat cells (numbers/mm^2^ bone marrow). Fields were taken from distal femur sections of 6-month-old (C57BL/6J and *ob/ob*) and 14-month-old mice and calculated using ImagePro software. Data are mean ± SE of 6 to 10 mice per age group. * *P*< 0.05, ** P< 0.001 HFD vs. chow diet and *ob/ob* vs chow-fed 6-month old. P value was calculated by two tailed T-test.

To compare the responsiveness of bone marrow adipocytes to a HFD and to explore whether age impacts the response of bone marrow and epididymal adipocytes to a HFD, 3- month-old and 11 month-old C57BL6/J mice were fed a HFD containing 60% calories from fat for 12 weeks prior to sacrifice at 6 and 14 months. For comparison, bone marrow and epididymal adipocytes from 6-month-old *ob/ob* mice maintained on standard chow diet were also studied. Table **1** depicts several metabolic parameters and circulating adipocytokines of the animals. The metabolic parameters show the expected negative effects of a HFD in mice. The HFD results in a similar degree of weight gain, hyperinsulinemia, and hyperglycemia at 6 and 14 months; however, there are differential effects on several parameters. For instance, leptin tends to increase with the HFD in 6, but not 14, month-old, whereas adiponectin decreases with the HFD in 14, but not 6, month-old mice (Table **1**). Circulating osteocalcin concentrations are unaffected by diet (though diminished with age); however, RANKL is decreased and osteoprotegerin is increased by the HFD at both 6- and 14-month-old (Table **1**). The HFD is accompanied by a significant increase in fat infiltration into the bone marrow in 6-month-old mice (Figure **1**); however, we were surprised to find that there was no further increase in bone marrow fat infiltration in 14-month old mice with HFD beyond that observed with age alone, which was 2-fold higher than that observed with HFD in 6 month-old mice. *Ob/ob* mice exhibit severe obesity, hyperinsulinemia, hyperglycemia, elevated resistin and adiponectin concentrations compared with 6-month old C57BL/6J standard chow fed mice. Circulating osteocalcin is significantly reduced in *ob/ob* compared to lean mice. RANKL and osteoprotegerin are altered in a similar manner in both DIO and *ob/ob* mice compared to lean mice. Interestingly, fat infiltration into the bone marrow of 6-month *ob/ob* mice was similar to that observed in 14-month old wild-type mice.

**Table 1:** Metabolic parameters in obese mice.

		**6-month**		**14-month**		**6-month**
**Parameter**	**Diet**	**LF**	**HF**	**LF**	**HF**	***Ob/ob***
	Weight (g)	35.0 ± 1.2	49.8 ± 3.3#	41.8 ± 4.0	67.1 ± 2.5*	76.8 ± 1.5#
	Insulin (ng/mL)	0.57 ± 0.16	2.98 ± 0.6#	2.09 ± 0.44	5.15 ± 0.84*	6.46 ± 1.0#
	Glucose (mg/dL)	118.7 ± 12	302 ± 91#	192.7 ± 17	255 ± 63*	317 ± 41#
	Triglyceride (mg/dL)	80.3 ± 2.13	93.3 ± 7.3#	83.6 ± 3.8	89.3 ± 5.2	93.3 ± 4.9#
**Adipokines**						
	Leptin (ng/mL)	3.52 ± 0.77	11.6 ± 3.3#	20.7 ± 2.38	18.6 ± 2.38	0.07 ± 0.01#
	Resistin (ng/mL)	1.35 ± 0.17	2.32 ± 0.36#	1.85 ± 0.20	2.64 ± 0.30*	1.75 ± 0.19#
	Adiponectin (µg/mL)	2.90 ± 0.20	3.10 ± 0.18	5.66 ± 0.74	3.00 ± 0.29*	3.8 ± 0.94#
**Bone panel**						
	Osteocalcin (ng/mL)	54.4 ± 4.8	46.4 ± 8.8	31.8 ± 6.8	30.8 ± 7.0	42.0 ± 4.3#
	RANKL (ng/mL)	0.21 ± 0.03	0.08 ± 0.03#	0.17 ± 0.02	0.05 ± 0.01*	0.01 ± 0.00#
	Osteoprotegerin(ng/mL)	2.28 ± 0.40	3.9 ± 0.03#	1.92 ± 0.17	4.92 ± 0.05*	5.82 ± 0.08#

Data are mean ± SE of 6 to 10 mice per age group. Body weights were determined at time of necropsy. Serum metabolites were measured by Luminex and determined in the fed state. LF, low fat diet; HF, high fat diet. *P< 0.05 vs. low fat diet 14-month old; #*P*< 0.05 vs. low fat diet 6-month old.

### Global characteristics of gene expression in bone marrow and epididymal adipocytes

**Figure 2:**
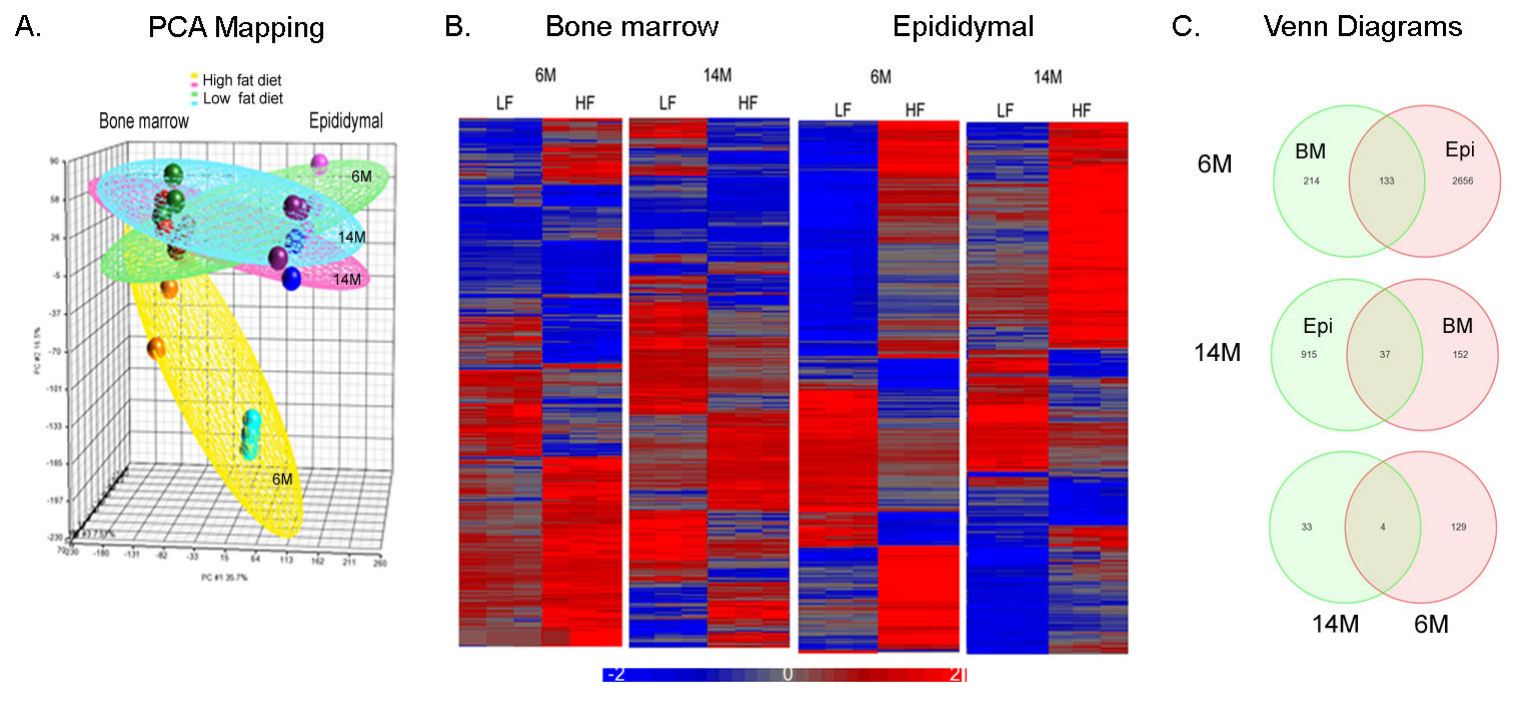
Diet induced obesity-mediated differential gene expression in bone marrow and epididymal adipocytes. Bone marrow and epididymal adipocytes were isolated from the same mice (age 6 months, 14 months fed standard chow or high fat diet (HFD)). Cells from several animals were pooled to yield sufficient sample to analyze three arrays for each age and diet group and both adipocyte populations. A total of 24 arrays were included in this analysis. (a) Principal component analysis of array data before normalization showing clustering of bone marrow adipocytes as separate from epididymal adipocytes. (b) Supervised hierarchical cluster analysis of diet-induced obesity genes in 6- and 14-month-old in bone marrow and epididymal adipocytes. (c) Venn diagram of overlapped genes induced by a HFD in 6- and 14-month-old in bone marrow and epididymal adipocytes.

We analyzed the differential expression of adipocyte genes in epididymal and bone marrow adipocytes from mice fed either standard chow or a HFD. Principal component analysis reveals a visible separation between bone marrow and epididymal adipocytes (Figure **2A**). In epididymal adipocytes, there is a clear separation between standard chow and HFD in 6-month-old mice. This clustering represents the overall expression patterns, but does not provide the interpretation of expression of individual genes. Figure **2B** shows the clustering of gene expression that is altered with the HFD. The HFD causes alterations in gene expression in both adipocyte depots at 6 and 14 months, but more genes are altered in epididymal than bone marrow adipocytes at each age and younger mice display more gene changes than older animals. Analysis of genes based on functional category was performed to assess the gene expression differences with the HFD. In epididymal adipocytes, a total of 2789 (9.6%) of the 28853 well-characterized mouse genes are differentially expressed at 6 months following a HFD, whereas 952 genes are regulated at 14 months with the HFD. In bone marrow adipocytes, there are 347 genes altered by the HFD at 6 months and 189 genes changed at 14 months. This selection was based on fold change ± 2.0 with *p* < 0.05. As displayed in the Venn diagram in Figure **2C**, the vast majority of genes that change in response to a HFD occur in a depot specific fashion, but 133 genes are altered by a HFD in both depots at 6-month-old, whereas 37 genes are altered by a HFD in both depots at 14 months (Figure **2C**). These genes were assigned to biologically meaningful gene ontology (GO) categories. Genes whose expression was regulated by a HFD in epididymal and bone marrow adipocytes are primarily found in three categories: biological process, molecular function and cellular component (Table **2**). Subcategories of biological process include regulation of cell cycle, cell death, and cell differentiation and regulation of metabolic process, for instance carbohydrate and lipid metabolism, whereas molecular function includes genes associated with protein binding, enzyme activity and transcription regulator activity, and cellular component includes genes associated with the cell membrane, extracellular matrix, synapses and membrane bound organelles. The table displays the enrichment score and *p*-value in subcategories of the overall category of biological processes that are most relevant to adipocyte-specific functions, whereas the subcategories of cellular component and molecular function are not shown because the *p*-value of these overall categories did not reach statistical significance. As shown in Table **2**, lipid metabolic process comprised 41% of genes with a high enrichment score in the ontology functional analysis. A high value of enrichment score and diet score indicates that genes in the functional group are differentially expressed with respect to diet. We next loaded this set of genes differentially expressed in epididymal and bone marrow adipocytes into Ingenuity Pathway Analyses for further pathway analysis (see below).

**Table 2:** Gene ontologies with differentially expressed genes in response to high fat diet.

**Functional Group**	**Functional Subgroup**	Enrichment Score*	*P* value**	**Diet Score**#	**% genes in group present**
Biological process		2.54	0.05	7.80	36.33
	Cellular process	39.07	0.001	7.27	41.18
	Lipid metabolic	21.61	0.001	8.53	49.28
	Carbohydrate metabolic	4.21	0.01	8.77	42.80
	Establishment of localization	17.53	0.001	7.04	41.18
	Transport	12.99	0.001	6.94	41.28
	Immune system	9.54	0.001	7.82	48.16
	Cell Growth	5.21	0.01	7.33	46.85
	Developmental	3.86	0.02	7.17	38.11
	Fat cell differentiation	1.61	0.2	9.58	42.59
	White fat cell	0.35	0.7	9.92	33.33
	Brown fat cell	1.31	0.32	12.58	42.31
	Metabolic process	2.78	0.06	7.44	37.23
Cellular component		2.37	0.09	7.03	36.35
Molecular component		1.22	0.29	7.04	36.26

Listed are top functional groups of high fat diet induced genes (FDR less than <0.05). * A high value of enrichment score indicates that the functional group is over-represented in the gene list. ** *P* value is the - log *p*-value of a Chi-square test.

### Obesity-related gene expression in epididymal and bone marrow adipocytes

We next sought to identify genes affected by HFD-induced obesity within each adipocyte population. Genes affected by obesity were identified and a group of gene lists was generated. A total of 133 genes are altered by a HFD in both depots at 6-month-old, whereas 37 unique genes are altered by a HFD in both depots at 14 months. The top regulated adipocyte-specific filtered genes are listed in Tables **3**-**6** for each depot and each age. The analyses were performed by comparing HFD to standard chow in 6 and 14-month-old animals, respectively. As listed in Table **3**, SPP1 (secreted phophoprotein 1), ADAM8 (ADAM metallopeptidase domain 8), and IL7R (interleukin 7 receptor) are the adipose-filtered genes most highly up-regulated in epididymal adipocytes at 6 months, whereas adipsin is the most highly down-regulated. While several of the same genes were observed to change in epididymal adipocytes at 14 months with the HFD (Table **4**), for instance ADAM8 and cathepsin K, the magnitude of the changes differed. Thus, the most highly up-regulated, adipose-filtered genes in epididymal adipocytes at 14 months are FABP9 (fatty acid binding protein 9) and ubiquitin D, whereas CD163 is down-regulated the most. The response to the HFD differed in bone marrow adipocytes. At 6 months Kruppel-like factor 2 (KLF2), ras homolog B (RhoB) and KLF4 are the most highly up-regulated, whereas thrombin receptor like 2 (F2RL2) and IL-6 are the most highly down-regulated (Table **5**). The magnitude of the changes in gene expression with the HFD at 14 months in bone marrow adipocytes was lower than observed at 6 months, with IL10, ATP2B4, and SOX6 the most highly up-regulated and serum induced kinase (SNK) down-regulated the most (Table **6**). Table **7** shows the genes (unfiltered) whose expression changed in the same direction in response to the HFD in both fat depots, with the genes listed in descending order based on the changes observed in epididymal adipocytes. As is apparent from this table, the magnitude of changes in expression were much greater in epididymal than bone marrow adipocytes, with Itgax (integrin alpha X), CD300ld, Dhrs9 (dehyrogenase/reductase member 9) and predicted gene 1002 among the most highly up-regulated. Interestingly, the expression of no gene was up-regulated in both epididymal and bone marrow adipocytes at 14 months, whereas secreted frizzled related protein 4 (Sfrp4) and the niacin receptor 1 (Niacr1) were down-regulated the most by the HFD. Surprisingly, the expression of only 4 genes (chemokine C-C ligand 2, dehydrogenase/reductase 9, fibrinogen-like protein 2 and polo-like kinase 2) was consistently and significantly altered in both depots and at both ages by the HFD. These are listed in Table **8** **.**

**Table 3:** Epididymal adipocyte genes most highly up- and down-regulated by a high fat diet at 6 months of age.

**Symbol**	**Entrez Gene Name**	**Fold change**	***P* value**
SPP1	secreted phosphoprotein 1	27.9	1.44E-07
ADAM8	ADAM metallopeptidase domain 8	27.1	9.95E-11
IL7R	interleukin 7 receptor	25.5	5.54E-09
STAP1	signal transducing adaptor family member 1	21.7	1.07E-10
CTSK	cathepsin K	21.5	5.01E-10
FCER1G	Fc fragment of IgE, high affinity I, receptor for; gamma polypeptide	21.3	4.65E-11
CD180	CD180 molecule	18.8	7.12E-11
LAT2	linker for activation of T cells family, member 2	18.1	2.28E-12
DPEP2	dipeptidase 2	17.6	5.08E-09
FYB	FYN binding protein	15.6	1.66E-09
ATP6V0E2	ATPase, H+ transporting V0 subunit e2	-5.5	9.56E-09
C2	complement component 2	-5.5	8.68E-11
TERC	telomerase RNA component	-6.5	2.54E-11
ADRB3	adrenergic, beta-3-, receptor	-6.6	3.06E-11
PTX3	pentraxin 3, long	-7.5	2.44E-08
ACVR1C	activin A receptor, type IC	-7.6	4.02E-10
GSTA4	glutathione S-transferase alpha 4	-8.3	3.19E-10
MOGAT1	monoacylglycerol O-acyltransferase 1	-8.5	2.42E-08
AMY2A	amylase, alpha 2A (pancreatic)	-9.2	3.70E-09
CFD	complement factor D (adipsin)	-13.3	1.03E-12

The list includes the adipocyte genes that change with high fat diet feeding at 6 months of age in epididymal adipocytes. The set of regulated genes was selected on the basis of their expression in adipose cells according to the IPA knowledge-base. For each gene, the fold change value in gene expression was calculated between mean values in epididymal adipocytes in standard chow and high fat fed with fold change ± 2 and *p* <0.05. The 6-month-old fed with high fat diet were compared to 6-month-old fed with standard chow diet. The significance of differences was measured by two-way ANOVA.

**Table 4:** Epididymal adipocyte genes most highly up- and down-regulated by a high fat diet at 14 months of age.

**Symbol**	**Entrez Gene Name**	**Fold change**	***P* value**
FABP9	fatty acid binding protein 9, testis	18.4	3.75E-12
UBD	ubiquitin D	15.5	6.88E-11
UCP1	uncoupling protein 1	6.6	5.45E-10
GADD45B	growth arrest and DNA-damage-inducible, beta	4.8	5.32E-09
AKR1B7	aldo-keto reductase family 1, member B7	4.3	4.12E-10
ADAM8	ADAM metallopeptidase domain 8	3.8	8.22E-06
FGF10	fibroblast growth factor 10	3.5	1.13E-07
CTSK	cathepsin K	3.5	2.80E-05
GADD45G	growth arrest and DNA-damage-inducible, gamma	3.5	3.64E-10
RGS2	regulator of G-protein signaling 2, 24kDa	2.9	4.11E-05
FMO_2_	flavin containing monooxygenase 2	-5.7	1.18E-07
PENK	proenkephalin	-6.5	1.77E-11
IGFBP6	insulin-like growth factor binding protein 6	-6.8	4.10E-10
CCR2	chemokine (C-C motif) receptor 2	-6.9	2.53E-07
PLA2G2D	phospholipase A2, group IID	-6.9	3.68E-07
SULT1E1	sulfotransferase family 1E, estrogen-preferring, member 1	-6.9	0.009232
SFRP2	secreted frizzled-related protein 2	-12.8	1.42E-07
F13A1	coagulation factor XIII, A1 polypeptide	-15.2	8.92E-11
LYVE1	lymphatic vessel endothelial hyaluronan receptor 1	-17.2	6.89E-13
CD163	CD163 molecule	-22.4	6.35E-10

The list includes the adipocyte genes that change with high fat diet feeding at 14 months of age in epididymal adipocytes. The set of regulated genes was selected on the basis of their expression in adipose cells according to the IPA knowledge-base. For each gene, the fold change value in gene expression was calculated between mean values in epididymal adipocytes in standard chow and high fat fed with fold change ± 2 and *p* <0.05. The 14-month-old fed with high fat diet were compared to 14-month-old fed with standard chow diet. The significance of differences was measured by two-way ANOVA.

**Table 5:** Bone marrow adipocyte genes most highly up- and down-regulated by a high fat diet at 6 months of age.

**Symbol**	**Entrez Gene Name**	**Fold change**	***P* value**
KLF2	Krüppel-like factor 2 (lung)	5.9	9.56E-06
RHOB	ras homolog gene family, member B	4.9	5.15E-07
KLF4	Krüppel-like factor 4 (gut)	4.2	7.30E-05
PLK2	polo-like kinase 2 (Drosophila)	3.7	1.48E-07
SH2D1B	SH2 domain containing 1B	3.5	1.32E-08
ENO3	enolase 3 (beta, muscle)	3.5	2.88E-05
ADRB2	adrenergic, beta-2-, receptor, surface	3.1	1.46E-07
NLRP3	NLR family, pyrin domain containing 3	3.0	0.000121
DUSP2	dual specificity phosphatase 2	2.8	0.000697
EGR1	early growth response 1	2.8	0.004784
MT1E	metallothionein 1E	-2.7	0.000561
NRGN	neurogranin (protein kinase C substrate, RC3)	-2.7	2.98E-05
ITGA6	integrin, alpha 6	-2.8	0.000123
PLS1	plastin 1	-2.9	7.20E-05
PRKCA	protein kinase C, alpha	-3.1	1.66E-08
GUCY1B3	guanylate cyclase 1, soluble, beta 3	-3.8	1.07E-05
ITGA2B	integrin, alpha 2b	-3.8	2.94E-05
CYSLTR2	cysteinyl leukotriene receptor 2	-4.2	5.89E-05
IL6	interleukin 6 (interferon, beta 2)	-4.9	1.60E-06
F2RL2	coagulation factor II receptor-like 2	-8.2	5.89E-07

The list includes the adipocyte genes that change with high fat diet feeding at 6 months of age in bone marrow adipocytes. The set of regulated genes was selected on the basis of their expression in adipose cells according to the IPA knowledge-base. For each gene, the fold change value in gene expression was calculated between mean values in bone marrow adipocytes in standard chow and high fat fed with fold change ± 2 and *p* <0.05. The 6-month-old fed with high fat diet were compared to 6-month-old fed with standard chow diet. The significance of differences was measured by two-way ANOVA.

**Table 6:** Bone marrow adipocyte genes most highly up- and down-regulated by a high fat diet at 14 months of age.

**Symbol**	**Entrez Gene Name**	**Fold change**	***P* value**
IL10	interleukin 10	2.6	0.0003216
ATP2B4	ATPase, Ca++ transporting, plasma membrane 4	2.1	0.0026892
SOX6	SRY (sex determining region Y)-box 6	2.0	0.0023088
SH2D1B	SH2 domain containing 1B	1.9	0.0167878
ASNS	asparagine synthetase (glutamine-hydrolyzing)	1.8	0.0045472
GAB1	GRB2-associated binding protein 1	1.8	1.36E-05
ACP1	acid phosphatase 1, soluble	1.7	1.41E-06
ADCY6	adenylate cyclase 6	1.7	0.0004744
UCK2	uridine-cytidine kinase 2	1.7	0.0018952
PRNP	prion protein	1.7	0.0010340
TNF	tumor necrosis factor	-2.9	0.0031101
JUN	activator protein 1, AP-1, C-JUN, JUNC, v-Jun	-3.1	3.60E-07
NLRP3	cryopyrin, NLR family, pyrin domain containing 3	-3.2	6.27E-05
CD69	CD69 antigen, CLEC2C, VEA	-3.4	2.37E-06
EGR1	Early growth response 1, EGR, Krox-1, KROX-24,	-3.5	0.001048
CSF1	CSF1 isoform 1, Csfm, M-CSF, MCSF1	-3.7	1.49E-08
PTGS2	COX-2, inducible cyclooxygenase	-3.8	4.04E-07
PMAIP1	APR, NOXA	-3.9	1.49E-05
H1F0	histone H1-0	-4.0	3.81E-09
PLK2	polo-like kinase 2, serum inducible kinase	-4.9	1.23E-08

The list includes the adipocyte genes that change with high fat diet feeding at 14 months of age in bone marrow adipocytes. The set of regulated genes was selected on the basis of their expression in adipose cells according to the IPA knowledge-base. For each gene, the fold change value in gene expression was calculated between mean values in bone marrow adipocytes in standard chow and high fat fed with fold change ± 2 and *p* <0.05. The 14-month-old fed with high fat diet were compared to 14-month-old fed with standard chow diet. The significance of differences was measured by two-way ANOVA.

**Table 7:** Genes highly up- and down-regulated by a high fat diet that are common to both epididymal and bone marrow adipocytes.

**Symbol**	**Entrez Gene Name**	**Epididymal**		**Bone marrow**		
**6-month**		**Fold change**	***P* value**	**Fold change**		***P* value**
Itgax	integrin alpha X	42.1	6.82E-10	2.8	0.00119556	
Cd300ld	CD300 molecule-like family member d	29.8	1.12E-11	2.2	0.00040224	
Dhrs9	dehydrogenase/reductase member 9	22.9	2.46E-11	2.3	0.00017422	
Cd180	CD180 antigen	18.8	7.12E-11	2.3	0.00019608	
Cd300lb	CD300 antigen like family member B	18	7.69E-12	2.5	1.37E-05	
Tlr8	toll-like receptor 8	15.4	3.30E-11	2	0.00034929	
Nlrp3	NLR family, pyrin domain containing 3	13.9	4.25E-09	3	0.00012105	
Fcgr4	Fc receptor, IgG, low affinity IV	13.7	3.36E-08	3.4	0.00016374	
Clec4a3	C-type lectin domain family 4, member a3	13.4	6.05E-09	3.8	1.85E-05	
Gm10002	predicted gene 10002	8.2	0.00064	15.3	5.84E-05	
AI324046	expressed sequence AI324046	-2.4	0.00151	-4.3	1.51E-05	
Myl9	myosin, light polypeptide 9, regulatory	-2.5	0.00187	-4.4	2.14E-05	
Pf4	platelet factor 4	2.3	0.00302	-4.4	1.96E-05	
Slamf1	signaling lymphocytic activation molecule family member 1	-2.3	0.00029	-4.6	6.09E-07	
AU023871	expressed sequence AU023871	-2.6	0.0038	-6.4	8.61E-06	
Gp9	glycoprotein 9 (platelet)	-3.4	0.00013	-5.6	3.61E-06	
Peg10	paternally expressed 10	-3.4	1.94E-06	-5	6.64E-08	
U29423	cDNA sequence U29423	-4.5	1.72E-07	-6.3	1.55E-08	
Ighg	Immunoglobulin heavy chain (gamma)	-7.3	2.26E-05	-6.3	5.06E-05	
Serpina3b	serine (or cysteine) peptidase inhibitor	-8.9	1.98E-08	-4.9	9.41E-07	
**14-month**						
Plk2	polo-like kinase 2 (Drosophila)	-2.3	1.18E-04	-4.9	1.23E-08	
Ccl2	chemokine (C-C motif) ligand 2	-2.7	4.1E-05	-2.8	1.66E-04	
LOC100047070	similar to Human Ig rearranged gamma chai	-2.7	0.00013	-3.6	6.01E-05	
Atf3	activating transcription factor 3	-2.8	2.33E-06	-5.0	6.73E-09	
Gla	galactosidase, alpha	-2.9	2E-07	-2.1	7.86E-07	
Errfi1	ERBB receptor feedback inhibitor 1	-3.2	2.81E-08	-2.2	0.00046955	
Timd4	T-cell immunoglobulin and mucin domain containing 4	-3.5	3.3E-08	-2.5	9.36E-05	
Jun	Jun oncogene	-3.6	6.86E-05	-3.1	3.60E-07	
Clec4n	C-type lectin domain family 4, member n	-4.0	1.28E-06	-2.1	0.00050524	
Csprs	component of Sp100-rs	-5.4	1.2E-06	-2.3	1.34E-07	
Niacr1	Niacr1 // niacin receptor 1	-7.8	6.76E-09	-3.6	3.47E-09	
Sfrp4	secreted frizzled-related protein 4	-22.4	6.4E-10	-2.9	1.51E-04	

**Table 8:** Genes altered by a high fat diet in each adipose depot at each age.

		**6-month**				**14-month**			
		**Epididymal**		**Bone marrow**		**Epididymal**		**Bone marrow**	
**Symbol**	**Entrez Gene Name**	**Fold change**	***P* value**	**Fold change**	***P* value**	**Fold change**	***P* value**	**Fold change**	***P* value**
Ccl2	chemokine (C-C motif) ligand 2	2.1	0.003011	2.4	0.000799	2.2	0.002058	-2.8	0.000166
Dhrs9	dehydrogenase/ reductase (SDR family) member 9	22.9	2.46E-11	2.3	0.000174	2.9	1.43E-05	-2.4	0.000143
Fgl2	fibrinogen-like protein 2	4.1	7.87E-09	2.1	1.29E-05	-3.5	3.31E-08	-2.6	9.23E-07
Plk2	polo-like kinase 2	11.5	4.65E-11	3.7	1.48E-07	2.4	1.84E-05	-4.9	1.23E-08

Tables **9** and **10** show the most highly up- and down-regulated genes with age while on a high fat diet in epididymal and bone marrow adipocytes, respectively. The analyses compared the gene expression in epididymal and bone marrow adipocytes from 14-months old HFD to 6-months old HFD mice. AMY2A (amylase, alpha 2A), TERC (telomerase RNA component) and CFD (adipsin) increased 4.6 to 5.3-fold in response to aging while on a high fat diet, whereas IL10 (interleukin 10) was down-regulated 19.4-fold in epididymal adipocytes with age. Interestingly, while IL10 increased 4.7-fold at 14 months in bone marrow adipocytes, KLF2 and KLF4 were the most down-regulated genes in bone marrow adipocytes with age (-9.4 and -10-fold), and were similarly down-regulated with age in epididymal adipocytes in the setting of a high fat diet.

**Table 9:** Epididymal adipocyte genes most highly up- and down-regulated with age during a high fat diet.

**Symbol**	**Entrez Gene Name**	**Fold change**	***P* value**
AMY2A	amylase, alpha 2A (pancreatic)	5.3	1.90E-05
TERC	telomerase RNA component	5.3	2.90E-05
GPT	glutamic-pyruvate transaminase	4.6	2.70E-05
CFD	adipsin, Complement Factor D, Factor D, PFD	4.5	2.70E-05
PHGDH	phosphoglycerate dehydrogenase	4.0	1.00E-04
PEPCK1	phosphoenolpyruvate carboxykinase 1 (soluble)	3.9	1.70E-04
MOGAT1	monoacylglycerol O-acyltransferase 1	3.0	1.70E-04
HSD11B1	hydroxysteroid (11-beta) dehydrogenase 1	2.9	0.000081
VLDLR	very low density lipoprotein receptor	2.9	0.000462
PDE3B	phosphodiesterase 3B, cGMP-inhibited	2.6	0.000362
AGPAT9	1-acylglycerol-3-phosphate O-acyltransferase 9	2.6	0.00011
PLIN1	perilipin 1	2.6	0.00021
ACAT2	acetyl-CoA acetyltransferase 2	2.2	0.000121
PPARG	peroxisome proliferator-activated receptor gamma	1.6	0.000797
KLB	klotho beta	1.6	0.000361
SCD	stearoyl-CoA desaturase (delta-9-desaturase)	1.5	0.004184
KLF4	Krüppel-like factor 4 (gut)	-5.3	1.60E-04
SOCS3	suppressor of cytokine signaling 3	-5.4	1.90E-05
GPR65	G protein-coupled receptor 65	-5.4	1.90E-05
ICAM1	intercellular adhesion molecule 1	-5.9	2.90E-05
CCL13	chemokine (C-C motif) ligand 13	-5.9	2.90E-05
SPP1	secreted phosphoprotein 1	-6.1	1.80E-05
IL1RN	interleukin 1 receptor antagonist	-6.2	1.90E-05
FGL2	fibrinogen-like 2	-6.6	1.70E-05
CD14	CD14 molecule	-8.2	1.67E-07
KLF2	Krüppel-like factor 2 (lung)	-8.7	1.06E-08
IL10	interleukin 10	-19.4	5.06E-09

The list includes the adipocyte genes that change with age in epididymal adipocytes on a high fat diet. The set of regulated genes was selected on the basis of their expression in adipose cells according to the IPA knowledge-base. For each gene, the fold change value in gene expression was calculated between mean values in epididymal adipocytes at 14 months compared with 6 months on a high fat diet.

**Table 10:** Bone marrow adipocyte genes most highly up- and down-regulated with age during a high fat diet.

**Symbol**	**Entrez Gene Name**	**Fold change**	***P* value**
P2RY10	purinergic receptor P2Y, G-protein coupled, 10	4.829	3.15E-04
IL10	interleukin 10	4.753	7.56E-03
CCR9	chemokine (C-C motif) receptor 9	4.349	1.30E-05
NR4A3	nuclear receptor subfamily 4, group A, member 3	4.043	2.32E-05
MYO1E	myosin IE	4.010	3.48E-05
GOT1	glutamic-oxaloacetic transaminase 1, soluble	3.693	2.88E-05
PDE3B	phosphodiesterase 3B, cGMP-inhibited	3.378	1.46E-05
DGAT1	diacylglycerol O-acyltransferase 1	2.011	0.000121
IL6	interleukin 6 (interferon, beta 2)	2.481	0.000697
SCD	stearoyl-CoA desaturase (delta-9-desaturase)	1.752	0.004784
PLIN1	perilipin 1	1.648	0.000561
FGL2	fibrinogen-like 2	-3.401	2.98E-05
GPR109A	G protein-coupled receptor 109A	-3.668	0.000123
RGS3	regulator of G-protein signaling 3	-3.894	7.20E-05
COL1A2	collagen, type I, alpha 2	-4.218	3.66E-06
CCL13	chemokine (C-C motif) ligand 13	-7.164	2.07E-06
JUN	jun proto-oncogene	-7.829	6.94E-08
EGR1	early growth response 1	-9.052	5.75E-08
KLF4	Krüppel-like factor 4 (gut)	-9.468	1.61E-07
KLF2	Krüppel-like factor 2 (lung)	-9.958	5.70E-07

The list includes the adipocyte genes that change with age in bone marrow adipocytes on a high fat diet. The set of regulated genes was selected on the basis of their expression in adipose cells according to the IPA knowledge-base. For each gene, the fold change value in gene expression was calculated between mean values in bone marrow adipocytes at 14 months compared with 6 months on a high fat diet.

**Figure 3:**
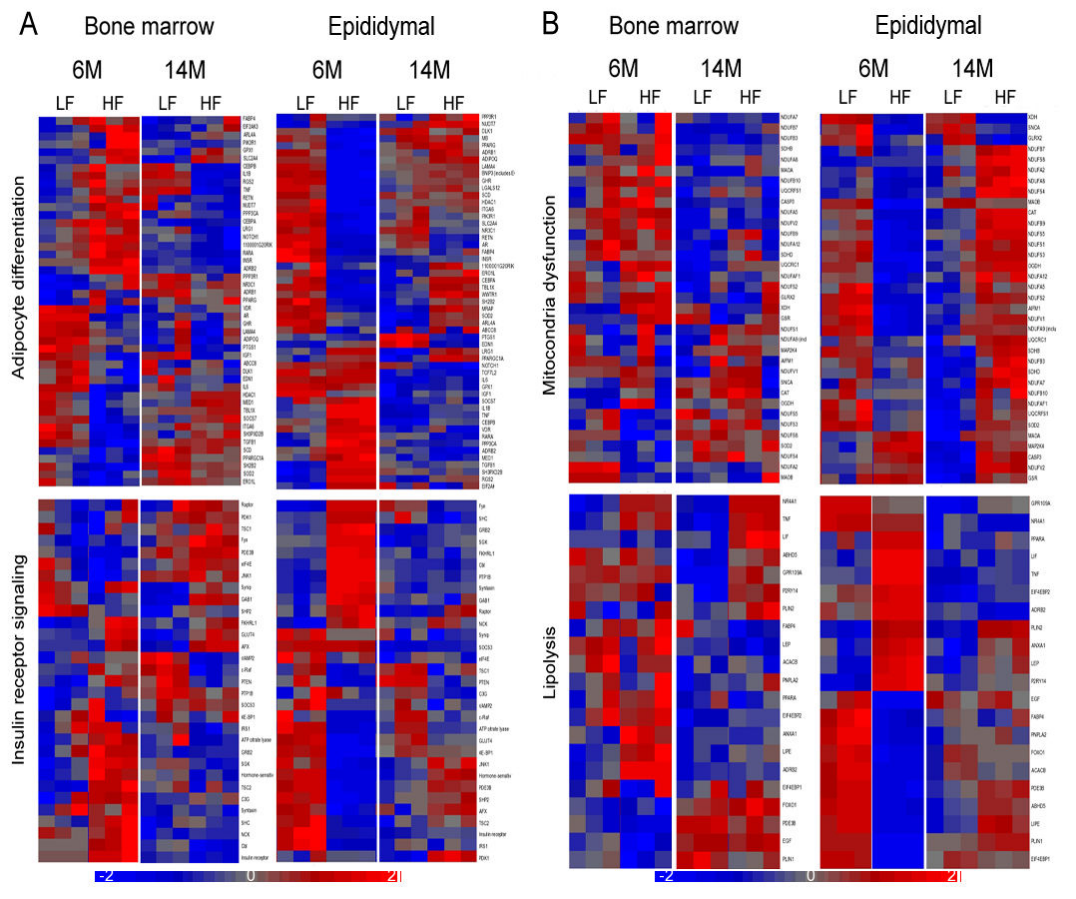
Heat maps of adipocyte-specific pathways in bone marrow and epididymal adipocytes in response to a high fat diet. (a) Clustering of genes involved in adipocyte differentiation (upper panel) and insulin receptor signaling (lower panel) with at least a 2-fold difference between standard chow and high fat diet in bone marrow and epididymal adipocytes. (b) Clustering of genes involved in decreased transmembrane potential of mitochondria (upper panel) and lipolysis (lower panel) with at least a 2-fold difference between standard chow and high fat diet in bone marrow and epididymal adipocytes.

Adipocyte-specific gene pathways, such as genes associated with differentiation, insulin receptor signaling and lipolysis, were generated using Ingenuity Pathway Analysis. Clustering of significantly altered genes involved with adipocyte-specific pathways in response to the HFD at each age is shown in Figure **3** **.** Figure **3A** indicates that adipocyte differentiation and insulin receptor signaling genes respond to the HFD in both fat depots at 6 months of age, whereas the responses are diminished in 14-month-old. Mitochondria function is affected in bone marrow adipocytes at 6-months by the HFD, whereas it was generally altered in epididymal adipocytes at 14-months (Figure **3B**). The genes involved with lipolysis are less impacted in response to the HFD in bone marrow than in epididymal adipocytes at 6-month-old (Figure **3B**).

### Functional analysis of differential gene expression in leptin deficient mice

**Figure 4:**
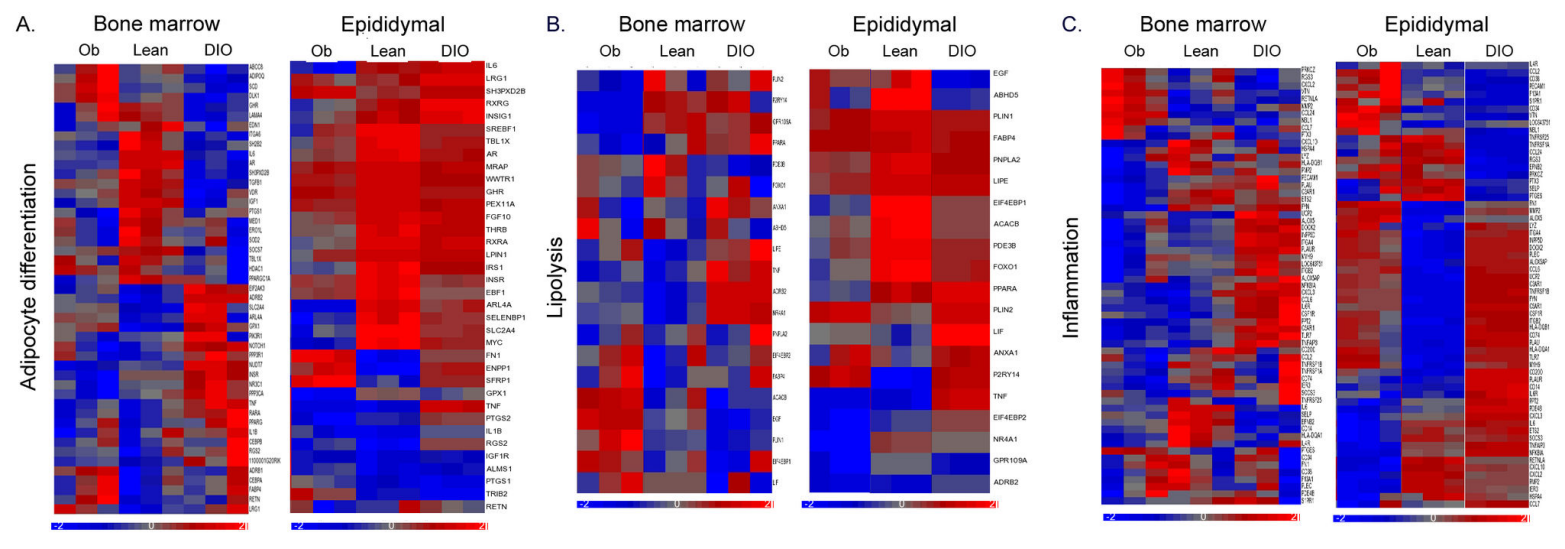
Heat maps of adipocyte-specific gene expression in bone marrow and epididymal adipocytes in 6-month-old diet-induced obese and *ob/ob* mice. (a) Clustering of genes involved in adipocyte differentiation in 6-month old diet-induced obese (DIO) and *ob/ob* compared with standard chow fed lean mice in bone marrow (left) and epididymal (right) adipocytes. (b) Clustering of genes involved in lipolysis in 6-month old DIO and *ob/ob* mice compared with standard chow fed lean mice in bone marrow and epididymal adipocytes. (c) Clustering of genes involved in inflammation in 6-month old DIO and *ob/ob* mice compared with standard chow fed lean mice in bone marrow and epididymal adipocytes.

We further explored the impact of obesity on gene expression in epididymal and bone marrow adipose depots by comparing *ob/ob* mice to DIO and lean mice at 6 months of age. In general, genes involved with adipocyte differentiation are suppressed to a greater extent in *ob/ob* mice than in DIO mice in both epididymal and bone marrow adipose depots (Figure **4A**). In bone marrow adipocytes, there are a few genes that are decreased in *ob/ob* but increased in DIO. These include ADRB2, EIF2AE1, NOTCH1, INSR, PPARγ and TNFα (Figure **4A**). As shown in Figure **4B**, genes involved in lipolysis are decreased in *ob/ob* and DIO mice; however, the response is smaller in bone marrow compared to epididymal adipocytes in both obese models. Genes associated with lipid droplets and lipolysis, such as PLIN2 and GPR109A, are decreased in bone marrow adipocytes in *ob/ob* but increased in DIO, whereas PLIN1 is increased in *ob/ob* but decreased in DIO. In contrast, PLIN1, PLIN2 and GPR109A are all altered in the same direction in epididymal adipocytes (Figure **4B**). Inflammatory response genes are similarly increased in both *ob/ob* and DIO mice in epididymal adipocytes, but DIO is associated with greater increases in inflammatory response genes in bone marrow adipocytes than *ob/ob* (Figure **4C**). These genes include IL6R, CCL2, and TNFα. However, some genes are increased in *ob/ob* compared to lean and DIO. These genes include PKCzeta, RGS3, CXCL2, RETNLA and MMP2. Interestingly, IL6 and IL6R are decreased in *ob/ob* in both fat depots, whereas they are altered in an opposite direction in DIO. Tables **11** and **12** show the adipocyte genes displaying the greatest differences in expression in *ob/ob* compared with 6-months HFD mice for epididymal and bone marrow, respectively. IL10 and TNF are the most reduced genes, whereas CCR2 and CXCL13 are increased the most in epididymal adipocytes in *ob/ob*. The response differed in bone marrow adipocytes where CIDEC and PLIN1 were higher and CXCR1 and SNORD37 were reduced the most in *ob/ob* mice.

**Table 11:** Epididymal adipocyte genes displaying greatest differences in leptin deficient mice compared with age matched high fat diet (HFD) fed mice.

**Symbol**	**Entrez Gene Name**	**Fold change**	***P* value**
CXCL13	chemokine (C-X-C motif) ligand 13	10.1	3.50E-05
CCR2	chemokine (C-C motif) receptor 2	9.6	8.11E-07
IL33	interleukin 33	7.2	6.84E-05
RTENLG	resistin like gamma	7.2	0.028536
COL3A1	collagen, type III, alpha 1	6.5	1.08E-08
TERC	telomerase RNA component	5.8	1.60E-07
SFRP2	secreted frizzled-related protein 2	3.8	2.57E-05
IGFBP6	insulin-like growth factor binding protein 6	3.7	1.24E-05
CCL12	chemokine (C-C motif) ligand 12	3.6	7.38E-05
CDKN1b	cyclin-dependent kinase inhibitor 1B	3.5	3.15E-05
CCL4	chemokine (C-C motif) ligand 4	-10.2	4.61E-06
FABP7	fatty acid binding protein 7	-10.3	1.19E-08
KLF4	Krüppel-like factor 4 (gut)	-10.7	1.90E-05
TNFAIP3	tumor necrosis factor, alpha-induced protein 3	-11.5	3.32E-09
NR4A1	nuclear receptor subfamily 4, group A, member 1	-11.7	7.73E-09
IL6	interleukin 6	-12.1	5.05E-06
CCRL2	chemokine (C-C motif) receptor-like 2	-14.0	2.44E-09
TNFSF9	tumor necrosis factor superfamily, member 9	-14.2	5.27E-09
TNF	tumor necrosis factor	-15.0	8.37E-09
IL10	interleukin 10	-18.5	1.24E-08

The list includes the adipocyte genes that differ in epididymal adipocytes between 6-month-old *ob/ob* mice on a normal chow diet and 6-month-old control mice on a high fat diet. The set of regulated genes was selected on the basis of their expression in adipose cells according to the IPA knowledge-base. For each gene, the fold change value in gene expression was calculated between mean values in *ob/ob* epididymal adipocytes at 6 months compared with 6-month-old controls on a high fat diet.

**Table 12:** Bone marrow adipocyte genes displaying greatest differences in leptin deficient mice compared with age matched high fat diet (HFD) fed mice.

**Symbol**	**Entrez Gene Name**	**Fold change**	***P* value**
CIDEC	cell death-inducing DFFA-like effector c	3.0	0.00166
PLIN1	perilipin 1	3.0	0.00074
PCK1	phosphoenolpyruvate carboxykinase 1 (soluble	2.7	0.00030
CDO1	cysteine dioxygenase, type I	2.7	0.0007
CFD	complement factor D (adipsin)	2.6	0.00186
SCD	stearoyl-CoA desaturase (delta-9-desaturase)	2.5	0.0006
APOC1	apolipoprotein C-I	2.2	0.00135
RETN	resistin	1.6	0.08256
CCR1	chemokine (C-C motif) receptor 1	-2.1	0.0007
GPR77	G protein-coupled receptor 77	-2.1	0.0003
KLF2	Krüppel-like factor 2 (lung)	-2.6	0.0103
CCL2	chemokine (C-C motif) ligand 2	-2.7	0.0017
PLK2	polo-like kinase 2	-2.8	0.0005
KLF6	Krüppel-like factor 6	-2.8	0.0004
TNFSF14	tumor necrosis factor superfamily, member 14	-2.9	0.00025
TLR8	toll-like receptor 8	-2.9	0.00049
CXCR1	chemokine (C-X-C motif) receptor 1	-3.0	0.000487
SNORD37	small nucleolar RNA, C/D box 37	-5.0	2.35E-05

The list includes the adipocyte genes that differ in bone marrow adipocytes between 6-month-old *ob/ob* mice on a normal chow diet and 6-month-old control mice on a high fat diet. The set of regulated genes was selected on the basis of their expression in adipose cells according to the IPA knowledge-base. For each gene, the fold change value in gene expression was calculated between mean values in *ob/ob* bone marrow adipocytes at 6 months compared with 6-month-old controls on a high fat diet.

## Discussion

Obesity is the most common metabolic abnormality found among adults in the industrialized world and is characterized by insulin resistance, though it is recognized that not all obese patients are insulin resistant. A variety of mechanisms have been proposed to explain the basis for insulin resistance as it relates to alterations in adipocyte functions, such as excessive fatty acid release (lipotoxicity), endoplasmic reticulum stress, disruption of mitochondrial function, or changes in secretion of adipocytokines. Adipocytes secrete an extensive list of factors [17–20] that can affect a number of systemic processes such as food intake, fatty acid oxidation, insulin sensitivity, and bone metabolism. It is widely accepted that obesity is characterized by evidence of a chronic, low-grade inflammatory process [21,22], as manifested by elevated circulating levels of acute phase proteins and cytokines, such as tumor necrosis factor α (TNFα), interleukin 1 (IL-1), and IL-6. In addition, examination of adipose tissue in these settings reveals increased expression of cytokines such as TNFα, IL-1 and IL-6, as well as and others. Moreover, it is apparent that there is heterogeneity in adipose depots and in the contribution and responses of various adipose depots to obesity and obesity-related metabolic alterations [23,24]. This study represents the first attempt to compare the response of primary bone marrow and epididymal adipocytes to a high fat diet at different ages by gene expression profiling.

Our results show that feeding a high fat diet to mice from age 3 months or from age 11 months for a total of 12 weeks causes a relatively similar degree of weight gain and metabolic deterioration, though there were some differences. Notably, there was a greater amount of weight gained in older mice on a high fat diet than younger mice, even though there was substantial weight gained with age on a normal chow diet. Though the high fat diet caused hyperinsulinemia and worsened hyperglycemia in both young and old mice, there was a greater deterioration in young mice owing to the development of hyperinsulinemia and hyperglycemia on a normal chow diet with aging. These alterations due to the high fat diet and the impact of age were also reflected in adipocytokine levels. For instance, circulating leptin levels increased in the young mice (6-month old) in parallel to the weight gain associated with the high fat diet, but leptin did not change with the high fat diet in older mice (14-month old) owing to the marked rise in leptin observed with age and increased weight in chow fed animals. Interestingly, bone related cytokines, RANKL (decreased) and osteoprotegerin (increased), were altered by high fat diet in both young and old mice. In contrast, osteocalcin was not affected by diet at either age, but declined with age. Although some investigators have reported that RANKL increases and osteocalcin decreases in response to a high fat diet [25,26], these inconsistencies are likely related to differences in diets (for instance, inclusion of cholate or cholesterol), ages and animal models utilized in these studies, since others have reported no effects of a high fat diet on osteocalcin [27]. Bone marrow adiposity was also influenced by diet and by age. Thus, the high fat diet resulted in increased numbers of adipocytes within the bone marrow in young mice (6-month old), as has been observed by other investigators [27–29], but did not change with the high fat diet in older mice (14-month old) owing to the marked increase in adiposity observed with age in chow fed animals. Thus, bone marrow adiposity is responsive to diet, but this response appears to be abrogated with age.

Our gene expression data indicate that nearly 10% (2789) of the genes expressed in epididymal adipocytes isolated from 6-month-old mice are statistically altered (≥2 fold) by feeding a high fat diet for 12 weeks. In comparison, only 347 genes expressed in bone marrow adipocytes isolated from the same 6-month-old mice are statistically altered (≥2 fold) by feeding a high fat diet for 12 weeks. At 14 months of age only 952 genes expressed in isolated epididymal adipocytes are statistically altered (≥2 fold) by feeding a high fat diet for 12 weeks, whereas only 189 genes expressed in isolated bone marrow adipocytes are altered by the high fat diet. Thus, adipocytes from both epididymal and bone marrow depots are responsive to diet, but the response is greater in epididymal than in bone marrow adipocytes. Moreover, this response is abrogated in both depots with age. The apparent decreased gene responsiveness of bone marrow, as compared with epididymal, adipocytes to a high fat diet is probably due to intrinsic differences in the gene expression patterns between these two adipose depots under basal conditions. In this regard, we have previously profiled the gene expression patterns in bone marrow and epididymal adipocytes [11]. Though sharing many features, 13% of genes are differentially expressed between bone marrow and epididymal adipocytes, with bone marrow adipocytes being characterized by low expression levels of genes involved in adipogenesis and high levels of genes associated with inflammation. In so far as a high fat diet is associated with an increased expression of inflammatory genes and with a decreased expression of lipogenic genes (at least in epididymal adipose tissue), it is likely that these changes are not observed in bone marrow adipocytes since this pattern of gene expression already is found in bone marrow adipocytes under normal chow conditions [11]. Likewise, the apparent decreased gene responsiveness of both bone marrow and epididymal adipocytes to a high fat diet with age is likely due to the fact that age is associated with increased expression of genes associated with inflammation and mitochondrial dysfunction in adipose cells [11], a pattern that is very similar to that induced by high fat feeding.

As compared with our previous report (11), the total number of genes whose expression was altered with age was attenuated in the setting of high fat feeding. For instance, there were 849 genes statistically increased (≥2 fold) in epididymal adipocytes at 14 months of age compared with 6 months old mice fed a normal chow diet (11), whereas there are only 122 genes (≥2 fold) altered with age at 14 months during high fat feeding. Interestingly, genes associated with lipid accumulation and storage (FASN, CFD, PLIN, Lep, SCD1) are increased greatly in response to aging while animals are on a chow diet (11), whereas they are not significantly altered in response to aging while on a HFD. In bone marrow, there were 312 genes increased (≥2 fold) at 14 months of age compared with 6 months old mice fed a normal chow diet (11), whereas there are only 17 genes significantly increased with age in the setting of high fat feeding. Similar to the response in epididymal depots, genes associated with immune function (IL7R, CD72) are the most regulated genes in response to age during high fat feeding. No genes were altered with age in epididymal or bone marrow adipocytes while on a high fat diet that we had not previously observed to be altered by age while on a chow diet (11). Thus, many of the changes in gene expression associated with increasing adiposity with aging appear to be attenuated in the setting of a high fat diet, whereas many of the alterations in gene expression associated with immune function appear to persist.

Several studies have previously reported on gene expression profiling of adipose tissue in either genetically or diet-induced obese mice and humans [10,30–37]. All of the studies involving mice have examined animals 6 months of age or younger, whereas we examined the response to obesity in both young and old mice. In addition, all previous studies have been conducted on RNA isolated from whole adipose tissue, whereas we profiled RNA expression from isolated adipose cells, thus decreasing the contribution to gene expression from non-adipose cells found within the fat depots. Nonetheless, our preparations of isolated adipose cells from both bone marrow and epididymal depots contained 10-17% cells that stained positively for CD11b [11], suggesting the presence of macrophages. The total number of genes whose expression was noted to be altered by obesity has varied among studies, ranging from 102 to 1658, depending on the methodologies utilized [10,30,32–37]. However, the overall pattern of gene changes observed has generally been similar, with increased expression of genes associated with inflammation, oxidative stress, and mitochondria, and with decreased expression of genes associated with lipogenesis and adipose differentiation. Previous comparisons of the effects of DIO on gene expression in various adipose depots showed the greatest number of changes in epididymal adipose tissue, followed by subcutaneous and then mesenteric fat [30]. This is similar to our observation of many fewer changes in gene expression in bone marrow compared with epididymal adipocytes with DIO. Interestingly, these authors noted an increased expression of markers of white blood cells (presumably monocyte/macrophages) in subcutaneous and mesenteric depots [30], suggesting that increased inflammation in these depots, similar to bone marrow adipocytes, dampened the response to the high fat diet.

It is interesting that, of the large numbers of genes altered by DIO in bone marrow and epididymal adipocytes at 6 and 14 months of age, most changes were depot specific. Thus, the expression of only 133 genes was altered by the high fat diet in both bone marrow and epididymal adipocytes at 6 months of age, whereas only 33 genes were altered in both depots by the diet at 14 months of age. Again, this is probably due to intrinsic differences in the gene expression patterns between these two adipose depots under basal conditions and to the impact of changes associated with normal aging in mice.

One of the striking differences in the gene expression between bone marrow and epididymal adipocytes in response to DIO was the observation that the high fat diet increased transcription factors controlling adipogenesis, such as PPARγ and its target genes, in bone marrow adipocytes in 6-month-old compared to lean mice. In contrast, the high fat diet was associated with a decrease in genes involved in adipogenesis, such as CFD (adipsin), PPARγ, FABP4 and GLUT4, in epididymal adipocytes from 6-month-old mice. These gene changes reflect the expansion of bone marrow adiposity by the high fat diet in 6-month old mice and are an example of how bone marrow adipocytes constitute a unique adipose depot and suggest how responses to a high fat diet could differentially alter adipose functions within the bone marrow that could potentially modify bone metabolism.

In addition to comparing the effects of a high fat diet on gene expression profiles of bone marrow and epididymal adipocytes, we also profiled bone marrow and epididymal adipocytes isolated from *ob/ob* mice with 6 month-old animals. *Ob/ob* mice fed a normal chow diet were heavier and more hyperinsulinemic than DIO mice at 6 months of age. Additionally, 6-month-old *ob/ob* mice have greater amounts of bone marrow adipocytes than DIO mice, displaying similar adipocyte infiltration as observed in 14-month-old animals. Overall gene expression was generally similar in epididymal adipocytes from *ob/ob* and DIO mice. For instance, inflammatory response genes were similarly increased in both *ob/ob* and DIO mice in epididymal adipocytes, but DIO was associated with greater increases in inflammatory response genes in bone marrow adipocytes than observed in *ob/ob*. Plin2 and GPR109A were decreased in bone marrow adipocytes in *ob/ob* but increased in DIO, whereas Plin1 was increased in *ob/ob* but decreased in DIO. In contrast, Plin1, Plin2 and GPR109A were all altered in the same direction in epididymal adipocytes. This differential expression of genes, particularly within bone marrow adipocytes, might reflect the importance of leptin signaling for bone metabolism [38].

In summary, bone marrow adipocytes, though responsive to a high fat diet, are less responsive to DIO than epididymal adipocytes and the response of both depots to DIO declines with age. This loss of responsiveness with age is likely due to age-associated changes in expression of genes related to adipogenesis, inflammation and mitochondrial function that are similar to and obscure the changes commonly associated with DIO. In conclusion, obesity affects age-associated alterations in gene expression in both epididymal and bone marrow adipocytes regardless of diet or genetic background.
